# Fully automated quantification of net water uptake in acute ischemic stroke using only non-contrast CT imaging

**DOI:** 10.1007/s00330-025-12238-0

**Published:** 2025-12-25

**Authors:** Thilo Sentker, Maximilian Nielsen, Susan Klapproth, André Kemmling, Michael H. Lev, Gabriel Broocks, René Werner

**Affiliations:** 1 Institute for Applied Medical Informatics, University Medical Center Hamburg-Eppendorf, Hamburg, Germany; 2 Institute of Computational Neuroscience, University Medical Center Hamburg-Eppendorf, Hamburg, Germany; 3 Department of Diagnostic and Interventional Neuroradiology, University Medical Center Hamburg-Eppendorf, Hamburg, Germany; 4 Department of Neuroradiology, University Hospital Marburg, Marburg, Germany; 5 Department of Radiology, Massachusetts General Hospital, Harvard Medical School, Boston, MA USA; 6 Department of Neuroradiology, HELIOS Medical Center Schwerin, University Campus of MSH Medical School Hamburg, Schwerin, Germany; 7 MSH Research, Development and Innovation gGmbH, MSH Medical University of Applied Sciences and Medical University, Hamburg, Germany

**Keywords:** Brain edema, Ischemic stroke, Non-contrast computed tomography, Image processing, Computer-assisted

## Abstract

**Objective:**

Estimating early lesion progression in ischemic stroke is essential for assessing thrombolytic treatment efficacy. While computed tomography perfusion (CTP) and diffusion-weighted imaging (DWI) are commonly used to determine irreversible tissue injury (core), their high costs and limited availability present challenges. An alternative is quantitative net water uptake (NWU) derived from non-contrast-enhanced CT (NCCT), a validated biomarker for predicting patient outcomes. However, current NWU measurements require time-consuming manual postprocessing, and automating this process remains challenging.

**Material and methods:**

This retrospective study introduces a fully automated method for NWU quantification using only NCCT images. The proposed image processing pipeline is based on expert-defined heuristics and voxel-wise NWU calculations, without employing deep learning-based components. The method was developed and validated on an in-house dataset of 185 patients (155 after exclusions) and externally tested on 51 patients (46 after exclusions). Performance was assessed by comparing computed lesion masks with expert-annotated core lesion masks from CTP (in-house) and DWI (external), using lesion detection rate, mean absolute NWU error, Dice similarity coefficient, and mean average precision.

**Results:**

Results demonstrated a high lesion detection rate of 94% (in-house) and 100% (external). The mean absolute NWU estimation error was 1.0% for in-house data and 1.5% for external data. Segmentation accuracy was moderate, with Dice similarity coefficients of 0.48 (in-house) and 0.47 (external).

**Conclusion:**

This method provides a practical and interpretable solution for NWU quantification, allowing stroke assessment directly from NCCT without reliance on additional imaging modalities.

**Key Points:**

***Question*** *Current approaches for quantifying early lesion progression in ischemic stroke rely on manual imaging-based NWU estimation, underscoring the need for an automated and reproducible method.*

***Findings*** *The method achieved high lesion detection accuracy and reliable net water uptake estimation, demonstrating its potential as a tool for automated stroke assessment.*

***Clinical relevance*** *This study presents an automated NCCT-based method for stroke assessment that enables net water uptake estimation directly from routinely acquired scans. The approach provides an interpretable alternative to methods requiring multimodal imaging, supporting broader clinical applicability.*

**Graphical Abstract:**

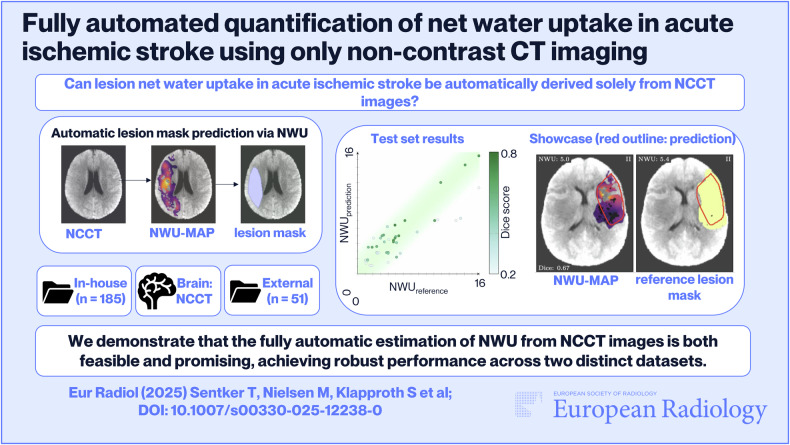

## Introduction

Infarct lesion volume is a key endpoint in clinical trials, typically assessed by comparing early follow-up imaging (24–72 h post-stroke) with baseline scans [1]. Computed tomography perfusion (CTP) is commonly used to estimate the ischemic core but has limitations, including restricted availability and a tendency to overestimate irreversible damage, potentially excluding treatable patients [2, 3]. This highlights the need for validated non-contrast CT (NCCT)-based biomarkers for routine clinical use.

Net water uptake (NWU), a prognostic biomarker for acute ischemic stroke (AIS), offers potential for refining treatment strategies [4]. NWU is calculated from NCCT hypodensity but typically requires infarct delineation using CTP or diffusion-weighted imaging (DWI). While DWI provides precise lesion boundaries, its use is limited by cost and availability [5]. CTP-based segmentation, on the other hand, lacks standardization and may overestimate infarct size due to subjective interpretation [6, 7].

Current NWU calculation relies on multiple imaging modalities and manual infarct delineation, which is time-consuming and operator-dependent [8]. An NCCT-only approach could minimize radiation (following the ALARA (as low as reasonably achievable) principle [9]), streamline workflows, and reduce treatment delays. Additionally, automated NWU quantification may help distinguish reversible from irreversible tissue damage, providing a practical and efficient alternative based solely on routinely acquired NCCT data.

Determining infarct volume from NCCT alone is challenging due to subtle hypodensity, limited contrast, and the often unclear visibility of core lesions in early stroke stages [10]. Kumar et al [11] estimate infarct volume from baseline CT but require CBF maps from CTP, while other NCCT-only approaches rely on semi-automatic deep learning (DL) methods that need expert refinement, increasing analysis time and complexity [12]. Even advanced DL techniques struggle with infarct segmentation due to data variability [13–15].

Inspired by Nowinski et al [16], who highlight the superiority of stroke imaging marker-based techniques over DL for detecting global density changes, we propose a method that integrates expert knowledge with local and global heuristics for automated NWU estimation. Avoiding deep learning and, therefore, any non-linear or non-transparent transformations, our pipeline employs data-efficient, interpretable, and computationally tractable techniques, reducing the risk of overfitting while ensuring reproducibility. The key contributions of this study are:

- A fully automated, explainable image processing approach for estimating NWU and infarct volumes from NCCT.
- Validation on an in-house dataset of 185 (155 after exclusions) patients, comparing NWU masks to expert-annotated core lesions from CTP.
- Evaluation on an external dataset, comparing NWU maps to reference DWI lesion masks.

These contributions build upon existing work to further advance ischemic stroke imaging and NWU estimation. Our findings demonstrate that NWU can be computed using NCCT images alone, providing a potential alternative to conventional stroke assessment methods that rely on CTP or DWI.

## Materials and methods

### Dataset

This retrospective study was conducted in accordance with ethical standards and was approved by the institutional review boards of the University Medical Center Hamburg-Eppendorf and the Massachusetts General Hospital Boston. Informed consent was waived as only retrospective and anonymized data were analyzed.

The first dataset, derived from 185 patients treated at the University Medical Center Hamburg-Eppendorf between 01/2019 and 05/2024, was used to develop and validate the fully automated NWU quantification pipeline. Patients included in this dataset had AIS in the middle cerebral artery territory, confirmed by large vessel occlusion on multimodal CT imaging performed within 6 h of symptom onset. The imaging protocol included NCCT, CT angiography and CTP scans. Patients were eligible if early ischemic lesions were visibly evident, defined as areas of reduced cerebral blood volume (CBV) within hypoperfused tissue, as identified on CTP scans using time-to-drain (TTD) maps. In cases where no CBV lesion was present, the region of interest was defined solely using TTD maps. Although the proposed NWU analysis pipeline relies exclusively on NCCT images, lesion masks were generated from CTP-derived TTD data, manually annotated by a medical expert with over 5 years of experience. These masks were used exclusively for algorithm development and optimization, with the computed NWU-based lesion map evaluated against these expert annotations. The NCCT images had a uniform spatial resolution of 1.00 × 1.00 × 1.00 mm^3^, with image dimensions ranging from [189–231] × [189–231] × [109–159] voxels. Following an initial inspection of the image data, 30 cases were excluded—18 due to poor image quality (e.g., metal implant artifacts, low signal-to-noise ratio) and 12 due to small lesion volume (< 1 mL)—resulting in 155 eligible cases for analysis.

The second, external data set, used for testing of the pipeline, originates from 51 patients treated at the Massachusetts General Hospital Boston, between 01/2007 and 09/2013. The ischemic core lesion was defined using DWI/apparent diffusion coefficient (ADC) maps with increased signal on DWI and reduced signal on ADC by clinical experts with more than 5 years of experience. Inclusion criteria required baseline NCCT scans with an onset-to-CT time of less than 6 h. The NCCT images had a spatial resolution of 0.43 × 0.43 × 5.00 mm^3^ and image dimensions ranged from 512 × 512 × [35–37] voxels. In this dataset, expert-annotated DWI lesion masks were used for algorithm testing; our algorithm was applied to compute NWU-based lesion maps, which were subsequently evaluated for correctness against the expert lesion masks. Cases with insufficient image quality due to artifacts and noise (*n* = 5) were excluded as unreliable for quantification, resulting in 46 usable datasets.

For both datasets, reference lesion masks delineated on TTD and DWI were mapped to the NCCT space using affine image registration (see “Automated NWU quantification” section for details). Core lesions were defined as volumes of severely ischemic tissue with uncertain viability, based on DWI/ADC or CBF.

### Automated NWU quantification

The NWU is defined as 1 minus the quotient of the average NCCT voxel intensity within the lesion mask and the corresponding region in the contralateral hemisphere:

1 $${NWU}=\left(1-\frac{\overline{{HU}\left(A\right)}}{\overline{{HU}\left(B\right)}}\right)\times 100$$

where $\overline{{HU}(A)}$ and $\overline{{HU}(B)}$ are the average Hounsfield Unit (HU) intensity values of the ischemic volume A and the contralateral normal volume B, respectively. Our approach to automated NWU analysis builds on this principle, leveraging the fact that infarcted regions exhibit high NWU values, while unaffected regions show lower values.

To eliminate the need for a predefined lesion mask manually extracted from, e.g., CTP data, we first compute NWU for small, overlapping subregions across the NCCT image, generating a voxel-wise NWU map for the entire brain. Next, the subregion with the highest NWU values is identified and encapsulated, and the NWU for that region is calculated. The source code will be made openly available at github.com/IPMI-ICNS-UKE/aNWU after acceptance.

**Fig. 1:**
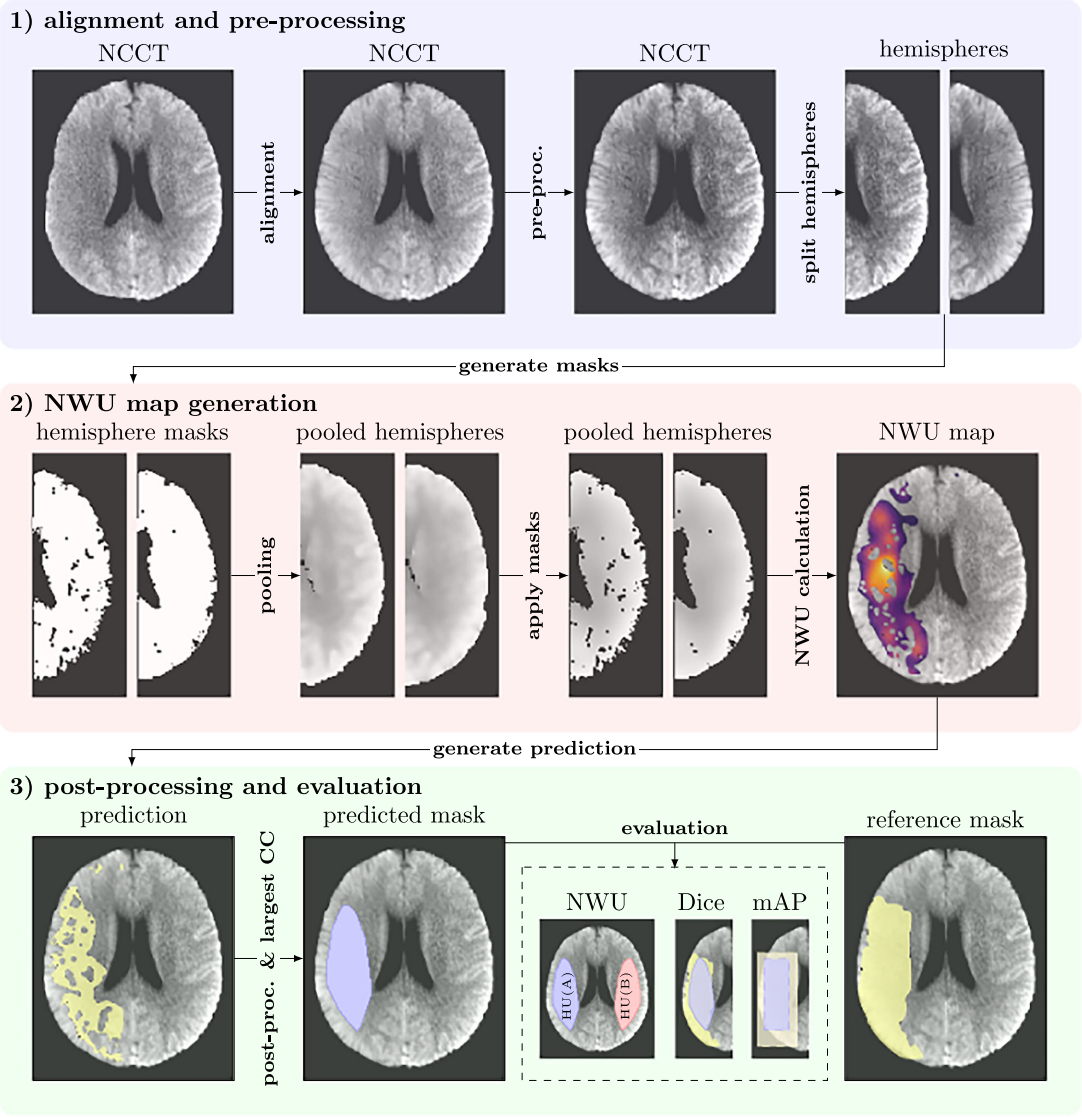
Proposed workflow for automated NWU quantification in NCCT data: Block 1—Affine image registration aligns the fixed (atlas) and moving (NCCT) images to the anterior commissure-posterior commissure (AC-PC) line, producing a symmetrical NCCT image. The image is then normalized and divided along the AC-PC line into brain hemispheres, with one hemisphere mirrored for spatial correspondence, enabling deformable registration between both hemispheres. Block 2—Hemisphere masks are generated, and corresponding regions are pooled and processed to compute the NWU map. Block 3—The NWU map is thresholded and post-processed to generate the lesion mask (largest connected component (CC)). For evaluation purposes, this mask is compared to the reference lesion mask (metrics: NWU; Dice; mean average precision (mAP))

The fully automated pipeline for estimating NWU from NCCT images consists of three main steps (a detailed formal description is provided in the Supplementary Materials):

(1) NCCT image pre-processing, (2) NWU map generation, and (3) NWU map post-processing, as illustrated in Fig. 1. The primary goal is to achieve accurate NWU quantification by utilizing brain hemispheric symmetry and imaging-based heuristics to delineate infarct regions. DL techniques were intentionally excluded to ensure transparency and explainability of the method developed.

Before processing, the NCCT images were resampled to an isotropic voxel size of 1 × 1 × 1 mm³ to ensure consistent spatial resolution across subjects and datasets. Subsequently, during the initial processing step (Fig. 1, subfigure 1), the images were aligned to the anterior commissure-posterior commissure (AC-PC) plane using affine registration with a proprietary CT brain atlas [17]. This alignment standardized spatial orientation across all images. Next, the AC-PC-aligned brain was split into left and right hemispheres, with the right hemisphere mirrored to match the orientation of the left. This mirroring enabled precise deformable registration between hemispheres using a publicly available, benchmarked toolbox (VROC, github.com/IPMI-ICNS-UKE/vroc) that ranked among the top performers in the MICCAI Learn2Reg challenges.

In step 2 (subfigure 2 of Fig. 1), non-brain regions are excluded by generating a mask for each hemisphere that includes all voxels with intensities larger than 0. Specific HU thresholds are applied to remove irrelevant areas such as bone and cerebrospinal fluid. Edge detection with a Sobel filter further refines the mask by excluding regions with strong edges that are unlikely to represent brain parenchyma. Following the idea of NWU calculation to analyze (infarcted) subregions of the brain, a 3D average pooling operation is applied voxel-wise to the masked hemispheres using a masked convolution kernel of size 11 × 11 × 11 mm^3^, which also helps to reduce noise and improve signal consistency. Regions in the processed image contributing fewer than 10% (i.e., 133 voxels) of the kernel volume are excluded. Finally, a voxel-wise NWU map is computed using Eq. 1 with the difference that $\overline{{HU}(A)}$ and $\overline{{HU}(B)}$ now refer to the average HU intensity values within corresponding sliding windows in the left and right hemispheres, respectively. Each voxel NWU value may therefore be interpreted as the NWU value of the surrounding brain tissue.

In step 3 (Fig. 1, subfigure 3), global NWU statistics were computed first, followed by Otsu’s method to define the threshold between background and foreground. The lower cutoff was chosen to exclude background noise while preserving subtle ischemic changes. Subsequently, the largest connected component in the NWU map was selected and subjected to post-processing operations, including erosion and dilation to refine lesion boundaries, followed by a flood fill operation to close small gaps and create a continuous lesion mask. For evaluation purposes, the resulting lesion mask is then compared to the respective reference lesion mask to evaluate the pipeline performance. To map CTP/TTD and DWI-based reference lesion masks onto the corresponding NCCT images, an affine atlas registration of the clinical image data used for lesion segmentation was performed. The reference masks were then transformed using the derived transformation parameters. For CTP/TTD data, the same brain atlas used for NCCT alignment was applied. For DWI data, an MR brain atlas had to be resampled and aligned to match the CT brain atlas, ensuring consistency in mapping the reference mask to the NCCT image data. The total relative NWU is subsequently calculated as defined in Eq. 1.

### Experiments and evaluation strategy

The experimental phase of this study consisted of two stages: (1) parameter optimization and algorithm validation, and (2) testing. In the first stage, key parameters and heuristics were selected, and the algorithm was validated using the in-house dataset. In the second stage, the proposed approach was tested on an independent external dataset to assess its generalizability and robustness.

In both stages, the estimated NWU was compared to the reference lesion mask-based NWU using the mean absolute error (MAE), which quantifies the average difference between computed and reference values. In addition, the median absolute error (median AE) and mean error (ME) were evaluated to provide a more robust assessment of variability and potential bias. For a subset of 15 cases of the external data set, annotations of an additional expert rater were used to quantify inter-reader agreement using the Pearson correlation coefficient. Additionally, a linear mixed-effects model that compares the model-computed NWU values with expert-based NWUs while accounting for repeated measures per scan through the inclusion of a random intercept was included. Segmentation and lesion detection performance were also evaluated. Lesion detection rate, defined as the proportion of cases where at least one voxel overlapped between the computed and reference segmentation masks, was assessed to measure hemisphere detection accuracy (HD acc.). For segmentation performance, the Dice similarity coefficient was used to quantify the overlap between computed and reference masks. Finally, the mean average precision (mAP) was calculated, where bounding boxes around masks with an intersection over union (IoU) greater than 0.5^3/2^ were considered correct detections. The threshold is derived from standard 2D image analysis practice and extended to 3D with the intention to balance sensitivity and specificity, ensuring robust detection performance [18]. All evaluation metrics were computed volumetrically across the entire supratentorial brain parenchyma.

## Results

Clinically relevant parameters for both datasets are listed in Table 1. Key parameters for our study, i.e., lesion extent (core/ASPECTS) and time from symptom onset to imaging, are comparable between the datasets, enabling a direct evaluation of our algorithm on both.

**Table 1:** Comparison of patient cohorts based on clinically relevant parameters

Patient characteristics	In-house validation cohort (*n* = 155)	External test cohort (*n* = 46)
Age (median [IQR])	76 [64; 83]	68 [54; 78]
Female (*n* [%])	94 [51]	20 [39]
NIHSS on admission (median [IQR])	15 [9; 19]	11 [8; 19]
Time symptom onset – admission in h (median [IQR])	3.1 [1.5; 5.9]	3.5 [2.2; 5.3]
ASPECTS (median [IQR])	8 [6; 9]	8 [7; 9]
Core lesion volume in mL (median [IQR])	10 [0; 32]	14 [5; 68]

**Fig. 2:**
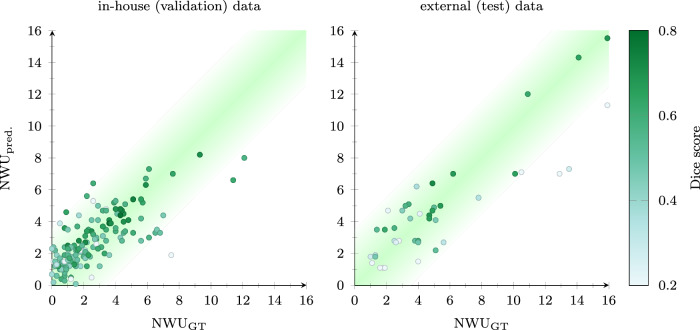
Reference NWU values (*x*-axis) compared to NWU values calculated using the computed lesion mask (*y*-axis) for in-house (left) and external data (right). Values are color-coded according to the respective Dice score between the reference and computed lesion masks (dark green: higher Dice, light green: lower Dice). The green diagonal represents an error band of ± 2%

The results demonstrate strong performance in infarct hemisphere detection. Table 2 summarizes the findings, with lesion detection rates of 0.94 for the in-house dataset and 1.00 for the external dataset, highlighting the reliability of the method in identifying infarcted regions across diverse patient populations. In Fig. 2, computed NWU values (on the *y*-axis) are compared to reference values (on the *x*-axis). For both the in-house dataset (left panel) and the external dataset (right panel), most values fall within a ± 2% confidence interval, indicating consistent agreement between computed and reference NWU values.

**Table 2:** Summary of evaluated metrics for in-house and external datasets

Stage	Dataset	*n*	HD acc.	NWU (%)			mAP	Dice
			(Dice > 0)	(MAE ± SD)	(ME ± SD)	(Median AE [IQR])	(> 0.5^3/2^)	(Median [IQR])
Val.	In-house	155	0.94	1.05 ± 1.00	0.21 ± 1.44	0.70 [0.40; 1.30]	0.48	0.48 [0.37; 0.55]
Test	External	46	1.00	1.48 ± 1.47	−0.50 ± 2.04	1.00 [0.40; 2.25]	0.57	0.47 [0.23; 0.60]

The method’s performance is assessed using hemisphere detection accuracy (HD acc.), mean absolute error (MAE), mean error (ME) and median absolute error (median AE) of the NWU, and two segmentation-based metrics: mean average precision (mAP) and Dice coefficient

On average, the MAE for NWU is 1.05% (ME: 0.21%, median AE: 0.70%) in the in-house dataset and 1.48% (ME: −0.50%, median AE: 1.00%) in the external dataset. Higher values in the external dataset may reflect differences in baseline reference mask generation methods (in-house: TTD from CTP imaging; external: DWI imaging), which could influence the segmentation accuracy. Inter-reader agreement was high, with a Pearson correlation of r = 0.98 between the two expert raters. The model-computed NWU values also showed strong correlation with each expert: r = 0.97 for both raters. The linear mixed-effects model with random intercepts per scan showed no significant difference between model-computed and rater-labeled NWU (β = 0.005, 95% CI: [−0.28, 1.24], *p* = 0.217).

The color coding of data points in Fig. 2 further illustrates segmentation performance: light green marks correspond to lower Dice similarity coefficients, indicating less overlap between computed and reference lesion masks, while dark green marks higher Dice coefficients, reflecting more accurate segmentation. Median Dice scores were comparable between the in-house and external datasets, with values of 0.48 (inter-quartile range [IQR]: 0.37–0.55) and 0.47 (IQR: 0.23–0.60), respectively. A Dice score greater than 0.3 was achieved in 84% of the in-house cases and 70% of the external cases. However, the external dataset showed better results than the in-house data in terms of mAP, with 57% of cases passing the predefined detection threshold (IoU > 0.5^3/2^) compared to 48% in the in-house data.

**Fig. 3:**
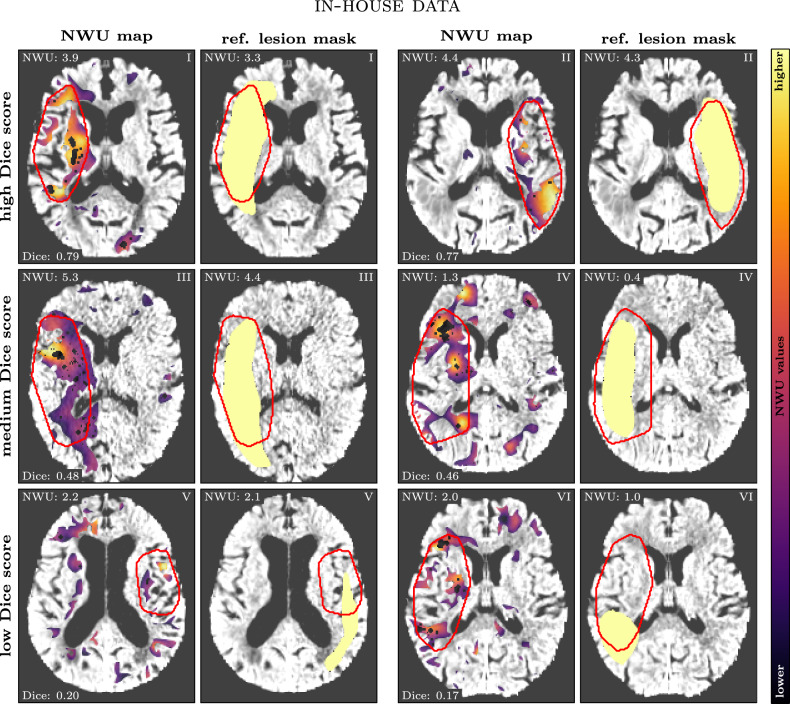
Six selected cases with varying Dice scores (two high, two medium, and two low; respective numbers are given in the bottom left corner of each comparison) for the in-house data set. For each case, the aligned brain NCCT image is shown twice: once overlaid with the NWU map (left; color-coded; dark: low NWU, bright: high NWU) and once overlaid with the reference lesion mask (right; light yellow). In both images, the computed lesion mask is outlined in red. Corresponding NWU and Dice values are displayed

**Fig. 4:**
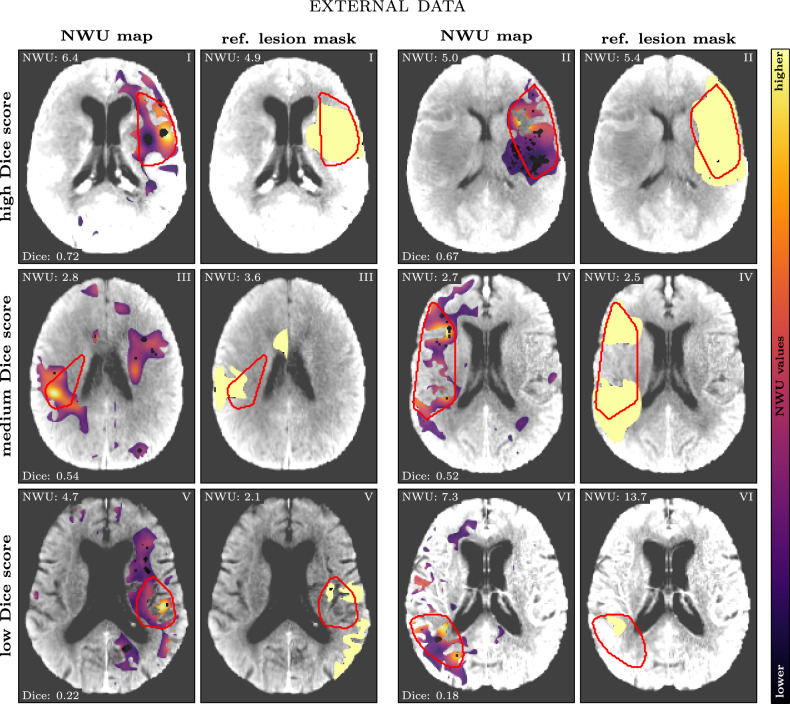
Six selected cases with varying Dice scores (two high, two medium, and two low; respective numbers are given in the bottom left corner of each comparison) for the external data set. For each case, the aligned brain NCCT image is shown twice: once overlaid with the NWU map (left; color-coded; dark: low NWU, bright: high NWU) and once overlaid with the reference lesion mask (right; light yellow). In both images, the computed lesion mask is outlined in red. Corresponding NWU and Dice values are displayed. Coronal views for these cases are provided in the Supplementary Materials

Visual inspection of the results highlights the potential of the method for clinical application. Figures 3 and 4 present six representative cases from the in-house and the external data set, showing NWU maps and computed/reference lesion masks. Cases were selected to span the full spectrum of agreement, i.e., two cases each with low, medium, and high Dice scores. In cases with high Dice similarity coefficients, the method accurately captured both the extent and location of infarct regions, closely aligning with reference annotations. Conversely, cases with lower Dice scores often involved smaller infarcts or regions with subtle hypoattenuation, which are inherently more challenging to delineate.

## Discussion

The results of this study demonstrate that fully automatic estimation of NWU from NCCT images is both feasible and promising, achieving robust performance across two different datasets. The lesion detection rate of 0.94 for the in-house dataset and 1.00 for the external dataset indicates that the proposed method reliably identifies infarcted hemispheres, even when using only baseline NCCT images acquired within 6 h of symptom onset. This is also reflected in the consistent quantification of the lesion NWU with low mean absolute errors compared to the ground truth NWU. Additionally, the approach maintains high interpretability and reproducibility by employing well-established, heuristic-based image processing techniques and linear operations rather than complex black-box DL models, making it well-suited for clinical implementation. To support uncertainty estimation, we included confidence intervals using a linear mixed-effects model, which confirmed high consistency between computed and rater-derived NWU values.

However, the segmentation performance, as evidenced by the Dice scores and mAP values, reveals areas for improvement. While the method performed well in detecting large infarcts and those with clear hypoattenuation, smaller lesions or regions with subtle ischemic changes proved more challenging to delineate accurately. The moderate overlap between computed and reference lesion masks suggests that additional refinement in the segmentation step is needed to capture the finer details of ischemic tissue. Nevertheless, compared to fully automated DL methods, our results are competitive [13–15], and our approach offers advantages: no training is required, the results are explainable, and the pipeline is fast (end-to-end processing time < 10 s per case). Hence, the method is potentially applicable in a real-world scenario for daily clinical practice and in prospective studies. The high lesion detection rate and the small NWU error, particularly on the external dataset, suggest that the method is adaptable to varying imaging protocols. The generalizability of the method across two clinical settings and cohorts is an encouraging sign for its potential as an applicable tool for AIS assessment. Furthermore, while NCCT offers a non-invasive and readily available imaging modality, the absence of perfusion information may limit the ability of the method to distinguish between early ischemic changes and normal anatomical variations in some cases. Moreover, the NWU maps, which form the backbone of our approach, provide clinicians with valuable insights into the segmentation process. These maps not only support the interpretation of the resulting segmentation but also help identify falsely detected regions or missed areas, thereby enhancing diagnostic confidence. Additionally, while our study focused on anterior circulation strokes, automated NWU quantification may also benefit posterior circulation cases, where imaging interpretation is often more complex.

Previous studies have introduced automated NWU estimation methods, including those by Kumar et al [11], Marcus et al [12], and Lu et al [19], which highlight the clinical relevance of NWU as a marker of ischemic damage. However, many of these approaches rely on DL models trained on relatively small datasets, require manual input or follow-up imaging, or provide region- or slice-level rather than volumetric estimates. In contrast, our method is fully automated, leverages expert-defined heuristics, and performs voxel-wise NWU estimation across the entire 3D supratentorial brain volume without the need for training data. This not only enhances reproducibility and interpretability but also makes the method more suitable for deployment in varied clinical environments.

Our study focused on patients imaged within 6 h of stroke onset, capturing early ischemic changes when NWU is often subtle. As a result, comparisons with TTD and DWI were exploratory and aimed at assessing NWU as a potential early marker of tissue injury. However, without follow-up imaging or defined thresholds for ADC, CBF, or TTD, the biological relevance of these comparisons remains uncertain. Furthermore, our relatively small cohort and the potential for selection bias limit the generalizability of our findings, as patients were selected based on specific clinical and imaging criteria. Another important limitation is the inability of NCCT-based NWU estimation to reliably differentiate infarct core from penumbra, as NWU primarily reflects hypoattenuation associated with irreversible injury and does not account for perfusion status. Although we explored heuristic strategies to approximate core-penumbra boundaries, these approaches proved insufficiently robust for clinical application and were not included in the final pipeline.

Future work could explore incorporating additional image processing techniques—such as DL-based segmentation or multi-modal fusion with minimal additional imaging data—to further improve accuracy. Additionally, applying our method to image data from other clinics would help assess its robustness and generalizability across different imaging protocols and patient populations. The overall performance of our method is already comparable to existing approaches that rely on additional imaging modalities, such as CTP [20], suggesting strong potential for clinical use in assessing AIS.

In conclusion, we presented a fully automatic method for NWU estimation in AIS using only NCCT images. The proposed method demonstrated robust performance, achieving high lesion detection rates and consistent NWU estimates compared with reference values. The ability to accurately estimate NWU and detect infarcted hemispheres using only standard NCCT images could be an advantage in clinical settings. Moreover, the method generalizes across different datasets and patient populations, underscoring its potential for widespread application in stroke assessment. With further development, this method could offer a valuable tool for rapid stroke assessment, enabling timely and effective clinical decision-making.

## Supplementary information

ELECTRONIC SUPPLEMENTARY MATERIAL

## Abbreviations

AC-PC: Anterior commissure-posterior commissure
ADC: Apparent diffusion coefficient
AIS: Acute ischemic stroke
ASPECTS: Alberta Stroke Programme Early CT Score
CT: Computed tomography
CTP: CT perfusion
DL: Deep learning
DWI: Diffusion-weighted imaging
IoU: Intersection over union
IQR: Interquartile range
MAE: Mean absolute error
mAP: mean average precision
ME: Mean error
Median AE: Median absolute error
NCCT: Non-contrast-enhanced CT
NWU: Net water uptake
TTD: Time-to-drain
