# Supplementary material for: Fully automated quantification of net water uptake in acute ischemic stroke using only non-contrast CT imaging
Source: Eur Radiol. 2025 Dec 25;36(6):4976–86. doi: 10.1007/s00330-025-12238-0 (PMC13212784; doi:10.1007/s00330-025-12238-0)
Supplement: Supplementary file 1 — ELECTRONIC SUPPLEMENTARY MATERIAL [file 330_2025_12238_MOESM1_ESM.pdf]

Fully Automated Quantification of Net Water Uptake in Acute Ischemic Stroke Using Only Non-Contrast CT Imaging

Supplementary Materials

Suppl. A. Net Water Uptake – Formal Definitions and Pipeline

Core definition

Let $HU(\mathbf{x})$ denote the Hounsfield Unit intensity at voxel coordinate $\mathbf{x} \in \Omega$ (image domain). For two corresponding spatial regions $A,B \subset \Omega$ the *Net Water Uptake* (NWU) of region A relative to region B is defined as

$$\mathrm{NWU}(A,B) \;=\; \left(1 - \frac{\overline{HU(A)}}{\overline{HU(B)}}\right) \times 100, \qquad \overline{HU(R)} \;=\; \frac{1}{|R|}\sum_{x \in R} H\,U(x), \qquad (1)$$

where $|R|$ is the number of voxels in region $R \subset \Omega$ and $\overline{HU(R)}$ its mean HU. NWU is expressed in percent.

Voxel-wise NWU map

Manual lesion delineation is time-consuming, prone to inter-observer variability, and limits reproducibility across studies. To overcome these drawbacks and enable fully automated, voxel-wise NWU estimation, we define for each voxel $\mathbf{x}$ an ischaemic-lesion-hemisphere neighborhood $\mathcal{N}_L(\boldsymbol{x})$ and a corresponding contralateral-normal neighborhood $\mathcal{N}_C(\boldsymbol{x})$ that are anatomically matched by the hemisphere registration procedure (see below). Let $\overline{HU(\mathcal{N}_L(x))}$ and $\overline{HU(\mathcal{N}_C(x))}$ be the mean HU within those neighborhoods. The voxel-wise NWU map is then

$$\mathrm{NWU}(x) \;=\; \left(1 - \frac{\overline{HU(\mathcal{N}_L(x))}}{\overline{HU(\mathcal{N}_C(x))}}\right) \times 100. \qquad (2)$$

Interpretation: $\mathrm{NWU}(\mathbf{x})$ is the NWU of the tissue surrounding voxel $\mathbf{x}$.

Neighborhood / pooling parameters

We implement $\mathcal{N}_L(x)$ and $\mathcal{N}_C(x)$ as cubic sliding windows of side length k voxels (odd integer) centered at the mapped coordinates. We further mask out voxels outside the plausible range (see below). Let

$$V_k \;=\; k^3$$

be the kernel (window) volume. To ensure sufficient data support, require that a window contributes at least

$$V_{min} = \lceil 0.10 \cdot V_k \rceil$$

voxels (i.e., at least 10% of the kernel volume). For $k = 11$ we have $V_k = 11^3 = 1331$ and $V_{min}\ in = \lceil 0.10 \cdot 1331 \rceil = 134$.

Pre-processing and hemisphere registration

To ensure consistent alignment between hemispheres and prepare the data for voxel-wise NWU computation, the input NCCT image $I(\mathbf{x})$ underwent the following preprocessing and registration steps:

1. Affine registration to a common CT brain atlas (AC–PC alignment): compute transform T_{ACPC} and apply to I to obtain I_{ACPC}.
2. Split I_{ACPC} into left (I_L) and right (I_R) hemispheres according to the mid-sagittal plane.
3. Mirror the right hemisphere to left orientation: $I_R^{\mathrm{mirr}} = M(I_R)$ where M denotes reflection across the midsagittal plane.
4. Perform deformable registration between I_L and I_R^{mirr} to obtain a dense mapping $T_{\mathrm{L \leftarrow R}}$ that maps coordinates in the mirrored right hemisphere to left-hemisphere coordinates (or vice-versa). Use this mapping to pair neighborhoods $\mathcal{N}_{\mathcal{L}}(\boldsymbol{x})$ and $\mathcal{N}_{\mathcal{C}}(\boldsymbol{x})$.

Masking and image conditioning Define an initial brain mask

$$\mathcal{B} = \{x \in \Omega : HU(x) > 0\}.$$

Further refine $\mathcal{B}$ by excluding voxels outside the plausible parenchyma range, defined by lower and upper HU thresholds (HU_{min},HU_{max}), to eliminate irrelevant regions. Compute a Sobel gradient magnitude image $G(\mathbf{x})$ and remove voxels with $G(\mathbf{x}) > \tau_e$ (strong edges), i.e.

$$\mathcal{B}_{\mathrm{refined}} = \mathcal{B} \setminus \{x : G(x) > \tau_e\}.$$

All subsequent computations are restricted to $\mathcal{B}_{\mathrm{refined}}$.

Computation of the NWU map (practical implementation)

For each voxel $\mathbf{x} \in \mathcal{B}_{\mathrm{refined}}$:

1. Construct $\mathcal{N}_L(\boldsymbol{x})$ as the $k \times k \times k$ cubic neighborhood in the left hemisphere centered at $\mathbf{x}$ (intersected with $\mathcal{B}_{\mathrm{refined}}$).
2. Map the center $\mathbf{x}$ to the contralateral hemisphere via $T_{\mathrm{L \leftarrow R}}$ and construct the anatomically corresponding neighborhood $\mathcal{N}_R(\boldsymbol{x})$ (also intersected with $\mathcal{B}_{\mathrm{refined}}$).
3. If $|\mathcal{N}_L(\boldsymbol{x})| < V_{\mathrm{min}}$ or $|\mathcal{N}_R(\boldsymbol{x})| < V_{\mathrm{min}}$ then mark NWU($\mathbf{x}$) as undefined (exclude).
4. Otherwise compute NWU($\mathbf{x}$) by Eq. (2).

Post-processing and lesion extraction

Let $\mathcal{M} = \{x : \mathrm{NWU}(x) \text{ is defined}\}$ and let $\mathrm{NWU}: \mathcal{M} \to \mathbb{R}$ be the computed map. Determine a global data set specific NWU background threshold τ_{NWU} through Otsu thresholding and threshold the NWU map at a chosen level τ_{NWU} to obtain a binary candidate map

$$\mathcal{C}(\tau_{\mathrm{NWU}}) = \{x \in \mathcal{M} : \mathrm{NWU}(x) \geq \tau_{\mathrm{NWU}}\}.$$

Extract connected components $\{C_j\}_{j=1}^{J}$ from $\mathcal{C}(\tau_{\mathrm{NWU}})$. For each component C_j compute:

$$|C_j|,\ \overline{\mathrm{NWU}}(C_j) = \frac{1}{|C_j|} \sum_{x \in C_j} NWU(x).$$

Select the lesion component according to a reproducible criterion, i.e.:

- **Default selection (mean NWU maximizer):**
$$j^* = \arg\max_{j:|C_j|\geq V_{\mathrm{sel}}} \overline{\mathrm{NWU}}\,(C_j),$$
where V_{sel} is a minimum allowable component volume (voxels). The final lesion mask is $L = C_{j^*}$.
- **Alternative (sum NWU / weighted):** maximize $|C_j| \cdot \overline{\mathrm{NWU}}(C_j)$ to favor larger components with high NWU.

Refine L using morphological operations: erosion $L \leftarrow \mathrm{erode}(L, S_{r_e})$, dilation $L \leftarrow \mathrm{dilate}(L, S_{r_d})$ and flood-fill closure to ensure topological continuity. Here S_r denotes a spherical (or cubic) structuring element of radius r voxels.

Reporting NWU

Once a final lesion mask L is obtained, compute regionwise NWU with Eq. (1) using $A = L$ and B as the contralateral homologous region (obtained via the hemisphere mapping). Report:

$$\mathrm{NWU}_{\mathrm{lesion}} = \mathrm{NWU}(L,\ \mathrm{contra}(L)).$$

Algorithm 1 Automated NWU estimation from NCCT

Require: NCCT image I, kernel size k, edge threshold τ_e, NWU threshold τ_{NWU}, selection volume V_{sel}

Ensure: Lesion mask L and lesion NWU $\mathrm{NWU}_{\mathrm{lesion}}$

$I_{\mathrm{ACPC}} \leftarrow \mathrm{AffineRegisterToAtlas}(I)$

Split I_{ACPC} into I_L, I_R; compute $I_R^{\mathrm{mirr}} \leftarrow \mathrm{Mirror}(I_R)$

$T_{\mathrm{L}\leftarrow\mathrm{R}} \leftarrow \mathrm{DeformableRegister}(I_L, I_R^{\mathrm{mirr}})$

$\mathcal{B} \leftarrow \{\boldsymbol{x}: HU(\boldsymbol{x}) > 0\}$; refine $\mathcal{B}$ by HU bounds and Sobel filtering

$V_k \leftarrow k^3$, $V_{\min} \leftarrow \lceil 0.10 V_k \rceil$

for each voxel $\mathbf{x} \in \mathcal{B}$ **do**

  Construct $\mathcal{N}_L(\boldsymbol{x})$ and $\mathcal{N}_C(\boldsymbol{x})$ via $T_{\mathrm{L}\leftarrow\mathrm{R}}$

  if $|\mathcal{N}_L(\boldsymbol{x})| < V_{\min}$ or $|\mathcal{N}_R(\boldsymbol{x})| < V_{\min}$ **then**

  Skip $\mathbf{x}$

  else

  Compute $\mathrm{NWU}(\mathbf{x})$ by Eq. (2)

  end if

end for

$\mathrm{C} \leftarrow \{\mathbf{x} : \mathrm{NWU}(\mathbf{x}) \geq \tau_{\mathrm{NWU}}\}$

Extract connected components $\{C_j\}$ from $\mathcal{C}$

$j^* \leftarrow \arg\max_{j:|C_j|\geq V_{\mathrm{sel}}} \overline{\mathrm{NWU}}\,(C_j)$

$L \leftarrow \mathrm{MorphologicalRefine}(C_{j^*})$

$\mathrm{NWU}_{\mathrm{lesion}} \leftarrow \mathrm{NWU}(L, \mathrm{contra}(L))$

return L, $\mathrm{NWU}_{\mathrm{lesion}}$

Mapping reference lesion masks for evaluation

When comparing to reference lesion masks derived from other modalities (CTP/TTD or DWI):

1. For each reference image compute the affine transform $T_{\text{ref}\rightarrow\text{NCCT}}$ that maps the reference image space into the NCCT AC–PC atlas space (the same CT atlas used above). Apply $T_{\text{ref}\rightarrow\text{NCCT}}$ to the reference mask.
2. If the reference mask originates from MR/DWI, resample the MR atlas to the CT atlas resolution prior to transforming the mask to preserve anatomical correspondence.
3. Evaluate overlap (e.g., Dice), volumetric agreement, and NWU differences between L and the transformed reference mask.

**Symbol**	**Definition**
$HU(x)$	HU intensity at voxel **x**
$\overline{HU(R)}$	Mean HU over region R
NWU(A, B)	Net Water Uptake of region A relative to B (Eq. 1)
NWU(**x**)	Voxel-wise NWU at **x** (Eq. 2)
A	Ischemic lesion region (final lesion mask)
B	Contralateral homologous (normal) region to A
$\mathcal{N}_L(\boldsymbol{x})$, $\mathcal{N}_R(\boldsymbol{x})$	Left / contralateral right local neighborhoods for voxel **x**
k	Side length (voxels) of cubic pooling window
V_k	Pooling window volume = k^3
Vmin	Minimum voxels required in a window ($\lceil 0.10 V_k \rceil$)
$\mathcal{B}$refined	Refined brain mask after HU thresholding and Sobel filtering
τ_e	Sobel gradient magnitude threshold (edge removal)
τNWU	Threshold on NWU for candidate lesion voxels (in percentage NWU)
C_j	j-th connected component in binarized NWU map
Vsel	Minimum component volume for selection
L	Final lesion mask after selection and morphological refinement

Table 1: Symbols and parameters used in the NWU pipeline.

Notation

Notes for reproducibility. Recommended default parameters used in our experiments:

$k = 5$, τ_e = (experiment-dependent; choose to remove skull edges)

$V_{\text{sel}} = 100$ voxels (example).

The implementation used in our study (source code) is released at github.com/IPMI-ICNS-UKE/aNWU.

Suppl. B. Additional Results

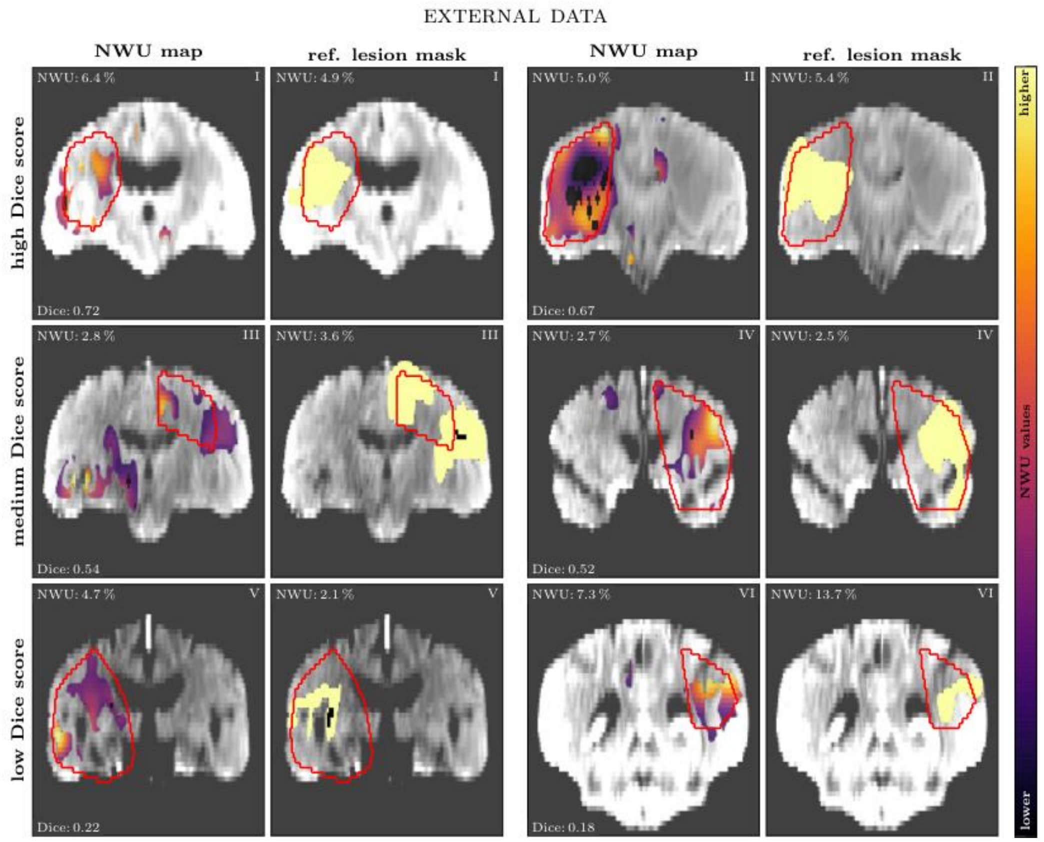

Figure 1: Coronal view of six selected cases with varying Dice scores (two high, two medium, and two low; respective numbers are given in the bottom left corner of each comparison) for the external data set. For each case, the aligned brain NCCT image is shown twice: once overlaid with the NWU map (left; color-coded; dark: low NWU, bright: high NWU) and once overlaid with the reference lesion mask (right; light yellow). In both images, the computed lesion mask is outlined in red. Corresponding NWU and Dice values are displayed.
